# Combination of immune checkpoint blockade with DNA cancer vaccine induces potent antitumor immunity against P815 mastocytoma

**DOI:** 10.1038/s41598-018-33933-7

**Published:** 2018-10-24

**Authors:** Alessandra Lopes, Kevin Vanvarenberg, Špela Kos, Sophie Lucas, Didier Colau, Benoît Van den Eynde, Véronique Préat, Gaëlle Vandermeulen

**Affiliations:** 1 Université Catholique de Louvain, Louvain Drug Research Institute, Advanced Drug Delivery and Biomaterials, Brussels, B-1200 Belgium; 2 Institute of Oncology Ljubljana, Department of Experimental Oncology, Zaloska 2, SI-1000 Ljubljana, Slovenia; 3 de Duve Institute, Université Catholique de Louvain, Brussels, B-1200 Belgium; 4 Ludwig Institute for Cancer Research, Brussels, B-1200 Belgium

## Abstract

DNA vaccination against cancer has become a promising strategy for inducing a specific and long-lasting antitumor immunity. However, DNA vaccines fail to generate potent immune responses when used as a single therapy. To enhance their activity into the tumor, a DNA vaccine against murine P815 mastocytoma was combined with antibodies directed against the immune checkpoints CTLA4 and PD1. The combination of these two strategies delayed tumor growth and enhanced specific antitumor immune cell infiltration in comparison to the corresponding single therapies. The combination also promoted IFNg, IL12 and granzyme B production in the tumor microenvironment and decreased the formation of liver metastasis in a very early phase of tumor development, enabling 90% survival. These results underline the complementarity of DNA vaccination and immune checkpoint blockers in inducing a potent immune response, by exploiting the generation of antigen-specific T cells by the vaccine and the ability of immune checkpoint blockers to enhance T cell activity and infiltration in the tumor. These findings suggest how and why a rational combination therapy can overcome the limits of DNA vaccination but could also allow responses to immune checkpoint blockers in a larger proportion of subjects.

## Introduction

Immunotherapy is an established approach to treat cancer based on the observation that the immune system can mount destructive responses against tumors. A major goal of immunotherapy is to develop a specific immune response against tumor-associated antigens (TAAs), which are derived from proteins that are specifically or preferentially expressed in tumor cells in comparison to non-transformed healthy cells^1^. DNA vaccines represent a good strategy to prime T cell responses against TAAs^2^. Furthermore, DNA vaccines can be used to deliver one or more antigens in their native conformation to develop a broad immune response^2^.

The amplitude of the developed immune responses is determined not only by the antigen recognition of T cells but also by co-stimulation/co-inhibition at the immunological synapse. Indeed, activated T cells express inhibitory receptors on their surface, such as CTLA4 and PD1, aiming at preventing autoimmunity^3^. These receptors are also responsible for the lack of effective antitumor immune responses in cancer patients, dampening T cell effector activity against tumor antigens. In particular, CTLA4 inhibits T-cell activation during the priming phase of immunity^4^. PD-1 transmits inhibitory signals into T cells after ligation with PD-1 ligands and promotes tolerance^5^. Antibodies blocking these molecules can increase the effector activity of tumor-specific T cells^6^. These antibodies, which are known as immune checkpoint blockers (ICBs), have already been approved by the FDA/EMA and used in the standard of care for different tumor types, such as melanoma and non-small-cell lung cancer^3,7^. However, ICBs have immune-related adverse effects, which commonly harm the gastrointestinal tract, endocrine glands, skin, and liver^7^. Moreover, ICBs are effective only in a minority of patients. In most advanced cancers, the response rate with anti-PD-1/PD-L1 monotherapy is only ~20%^8^, and the response rate with anti-CTLA4 is approximately 12%^3^, indicating the need for improvement. This low efficacy may be due to a lack of pre-existing tumor-associated T cell immunity.

The murine mastocytoma P815 tumor model was used in the present study. This model is characterized by the expression of different TAAs, particularly P815A, encoded by the *P1A* gene as previously described^9^. P815A shares many characteristics with human MAGE-type (melanoma antigen gene) tumor antigens^9^, suggesting P815 mastocytoma as a good preclinical tumor model for future applications in human medicine. We have previously developed a codon-optimized vaccine encoding P815A^10^. We demonstrated that the optimized vaccine increased antigen expression and activated innate immunity while retarding tumor growth in both preventive and therapeutic settings^10^. However, therapeutic vaccination delayed tumor growth but only slightly increased the survival of mice.

In this study, we aimed to generate a more potent immune response by combining DNA vaccination with ICBs. We hypothesized that this combination can improve the therapeutic efficacy of the DNA vaccine and increase the number of mice responding to ICB by “releasing the brakes” of T cell activity and by activating a higher number of antigen-specific T cells. We also evaluated the effects of the two strategies in the tumor microenvironment (TME) in an early phase of tumor development and metastasis formation that, until now, has been poorly explored.

## Results and Discussion

### The combination of pP1A vaccine and ICBs delayed tumor growth and increased mouse survival

**Figure 1:**
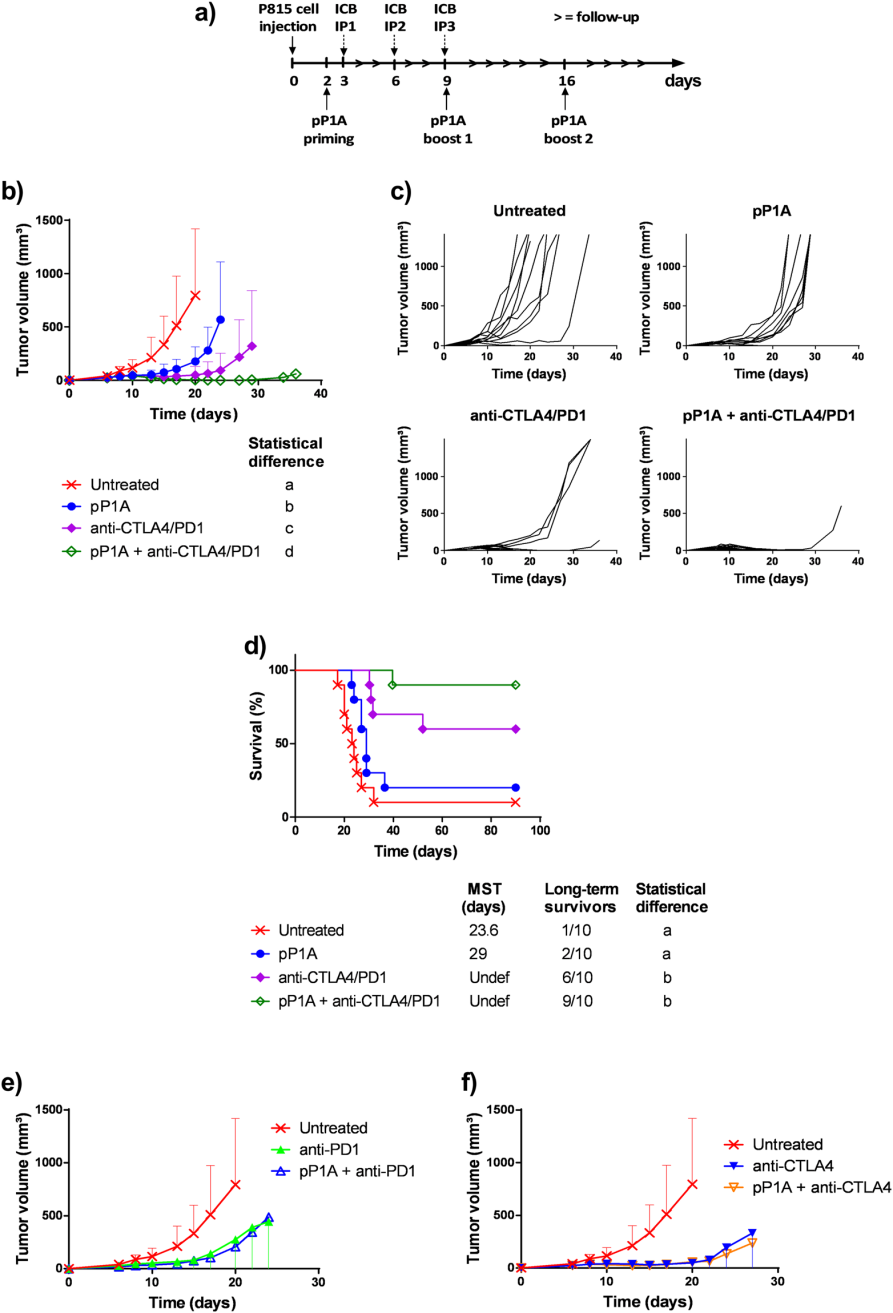
Therapeutic combination of pP1A and ICBs (anti-CTLA4/PD1). (**a**) Therapeutic vaccination protocol. ICBs were administered intraperitoneally (IP) 3 times every 3 days starting at day 3. The pP1A vaccine was administered 3 times weekly starting at day 2 by intramuscular electroporation. (**b**) Evolution of tumor volume (mm^3^) after P815 challenge as a function of time (days) (mean ± SD). All the groups were statistically compared to the others using two-way ANOVA, column factor (p < 0.05, n = 10). (**c**) Tumor growth in individual mice and for every group of mice (n = 10). (**d**) Survival curve representing the percentage of living mice (%) as a function of time (days). Statistical analysis using log-rank (Mantel-Cox) test (significant difference when p < 0.05, n = 10). MST = median survival time. (**e**,**f**) Measurement of tumor volume (mm^3^) after P815 challenge as a function of time for the anti-PD1 and pP1A + anti-PD1 groups (**d**) or for the anti-CTLA4 and pP1A + anti-CTLA4 groups (**e**) (days) (mean ± SD, n = 10). The absence of common letters in the statistical analysis (**a**–**d**) indicates statistically different results.

To assess the therapeutic efficacy of the combination of pP1A with ICBs, tumor-bearing mice were treated with pP1A alone or in combination with anti-CTLA4 and anti-PD1. The protocol is shown in Fig. 1a. Tumor growth was significantly slower for all the treatments compared to the untreated group and, importantly, was significantly slower in the group receiving pP1A in combination with ICBs than in the group receiving ICBs alone or pP1A alone (Fig. 1b). Indeed, tumors in the untreated group started to grow 8 days after tumor injection, and their growth was exponential. In the other groups, tumor volumes remained constant between day 8 and 13. Some of the tumors started to regrow after that period. In particular, pP1A alone delayed tumor growth, with the tumors starting to regrow at day 15 after a slight regression/plateau around day 10. The growth of tumors in individual mice is shown in Fig. 1c. Compared to the untreated mice, all the other mice survived longer (Fig. 1d). Survival was 20% or 60% when pP1A or ICB was used alone, respectively (Fig. 1d). Using the combination therapy of pP1A with ICBs, 90% of mice survived (Fig. 1d). However, the difference between ICBs and pP1A + ICBs was not statistically significant despite the higher survival trend observed in the group treated with the pP1A + ICB combination. The result obtained with pP1A alone confirmed our previous work on this tumor model, indicating the ability of a DNA vaccine to slowdown the tumor growth, but not to permit a complete and significant tumor rejection^10^. The combination of pP1A with ICB further decreased tumor growth even if the doses of ICBs used in this study were lower than others that employed the same therapeutic combination (100 µg compared to 200–250 µg per dose, per antibody)^3,11^. This encouraging result suggests the possibility to reduce the dose of ICB when they are administered in combination with DNA vaccination, to decrease the toxicities associated to these antibodies in human patients^12^.

The efficacy of anti-PD1 in our model may be due to the expression of *PD-L1* mRNA in P815 cells and in the untreated tumors (Supplementary Data 1). The rationale for the use of a second ICB is their additive effect on increasing mouse survival compared to the use of a single ICB^13,14^. Indeed, in the current experiment, the combination of pP1A with one ICB at a time was also tested by measuring the tumor growth in treated mice. No significant differences were observed between a single ICB used alone and one used in combination with the vaccine (Fig. 1e,f). Das *et al.*^15^ observed that the blockade of CTLA-4, PD-1, or the combination of the two leads to distinct genomic and functional signatures *in vivo* in purified human T cells and monocytes. Combination blockade induces non-overlapping changes in gene expression^15^. This complementarity justifies the high interest in using more than one ICB in preclinical and clinical studies^6,16–19^. In particular, the anti-CTLA4 antibody can enrich and amplify the T cell repertoire^20^, which is improved by priming with cancer vaccination, thereby inducing a proliferative signature to tumor-infiltrating T cells^21^. In contrast, the anti-PD1 antibody reactivates disabled intratumoral T cells and modulates their cytolytic function^21^. Furthermore, anti-PD1 therapy is more effective when tumor cells express *PD-L1*, as its expression is significantly associated with greater clinical response rates to anti-PD1 treatments^22^. Hence, the combination of ICBs and cancer vaccination may transform poorly immunogenic tumors into ‘inflamed’ tumors, which are more sensitive to treatments and the host immune response^6,21^. The rational of this effect could be the complementary mechanism of DNA vaccination and ICB. Vaccination can help the phase of antigen processing and presentation that is dampened during tumor development, while ICB can sustain T cell activation and trafficking into the tumor. Hence, ICB could enhance the infiltration and activity of antigen-specific T cells generated by DNA vaccination. To verify this hypothesis, an analysis of T cell infiltration in an early phase of tumor development was performed.

### Tumor infiltration by CD4 T cells was higher when mice were treated with a combination of pP1A vaccine and ICBs

**Figure 2:**
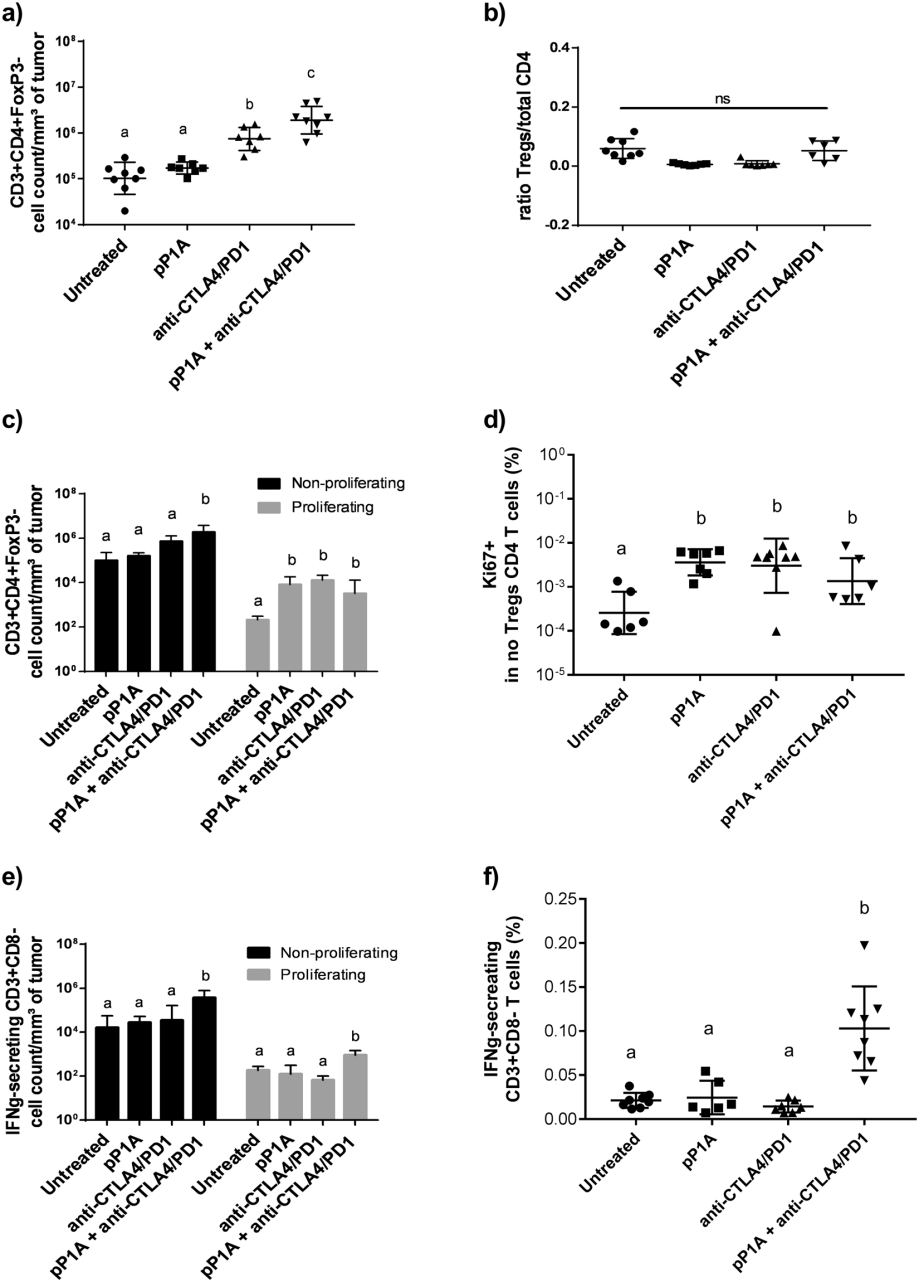
Evaluation of CD4 infiltration in tumors. (**a**) Number of non-Treg CD4 T cells (CD3 + CD4 + FoxP3−) per mm³ of tumor. (**b**) Ratio of Treg cells (CD3 + CD4 + FoxP3+) compared to the total number of non-Treg CD4 T cells. (**c**) Number of proliferating and non-proliferating non-Treg CD4 T cells per mm³ of tumor. (**d**) Percentage of proliferating non-Treg CD4 T cells per mm³ of tumor. (**e**) Number of IFNg-secreting CD3 + CD8− T cells per mm³ of tumor. In (**c**) and (**e**), two independent statistical analyses have been performed for non-proliferating and proliferating cells. (**f**) Percentage of IFNg-secreting CD3 + CD8− T cells per mm³ of tumor. All the results are expressed as the mean ± SD (n = 6–8) and were considered statistically significant when p < 0.05 (indicated by the absence of common superscript letters) according to one-way ANOVA.

To assess the early phase cellular immune response in the tumor microenvironment, CD4 T cell infiltration was evaluated in tumors resected 10 days after P815 injection. Single cell suspensions were analyzed via flow cytometry to detect CD3 + CD4 + FoxP3− T cells (non-Treg CD4 T cells) and CD3 + CD4 + FoxP3 + cells (Tregs). In general, a higher amount of non-Treg CD4 T cells  was detected in tumors of mice treated with ICBs and pP1A + ICBs (Fig. 2a), and the ratio of Tregs to total CD4 T cells was not different between the groups (Fig. 2b). This result means that the treatments did not reduce Treg infiltration in the early phase of tumor development. The reason of this result is not clear, as ICBs can reduce Treg infiltration in the TME according to the literature^23^. Our hypothesis is that the effects of ICBs on Treg depletion are visible in a later phase of tumor development. It would be interesting to follow the evolution of Treg infiltration and activity in the tumor microenvironment as a function of time. Nevertheless, there was a higher density of non-Treg CD4 T cells in the tumors of mice treated with ICBs. According to the literature, this effect may be principally due to the anti-CTLA4 antibody. The role of monoclonal antibodies that block inhibitory immune checkpoint molecules in enhancing T cell infiltration has been previously described^24,25^. Bengsch *et al*. found that disruption of the CTLA-4 interaction with CD80 using an anti-CTLA4 antibody induces CD4 T cell infiltration into tumors^26^. However, although the vaccine alone did not have any effect on CD4 T cell infiltration, its combination with ICBs further increased the number of non-Treg CD4 T cells into the tumor. In some preclinical models, cancer vaccines enhanced effector T-cell infiltration into tumors when combined with other therapies^27^. Furthermore, the group receiving the vaccination alone promoted a higher CD4-non Treg proliferation (Ki67 + cells) compared to the untreated group (Fig. 2c,d and Supplementary Data 2b). Ki67 is a nuclear protein associated to cell proliferation. It is present during all active phases of the cell cycle (G(1), S, G(2), and mitosis), but is absent from resting cells (G(0)), making it an excellent marker for determining the growth fraction of a given cell population^28^. This means that the vaccine can influence the proliferative capacity of infiltrated CD4 and increase the number of CD4-non Tregs inside the tumor, as observed in the combination group. Finally, CD3-positive cells were labeled with anti-IFNg antibodies to detect IFNg-secreting cells, and CD8 T cells were excluded from this analysis (Fig. 2e,f). This experiment clearly demonstrated that there is higher IFNg production from both proliferating and non-proliferating (Ki67- cells, cells in G(0) phase) non-CD8 T cells (including CD4 T cells) when the combination therapy is used. Currently, the important role of CD4 T cells in tumor regression is being elucidated^29^. In a clinical study of esophageal squamous cell carcinoma, increased CD4 T cell infiltration was significantly associated with longer overall survival^30^. In the P815 model, Rahir *et al*., demonstrated that the infiltration of CD8-specific T cells requires CD4 T cells^31^. For this reason, a growing number of cancer vaccines have been designed to contain at least one CD4 epitope^11,32–34^.

### CD8 T cell proliferation, activity and cytokine production were higher in the combination group

**Figure 3:**
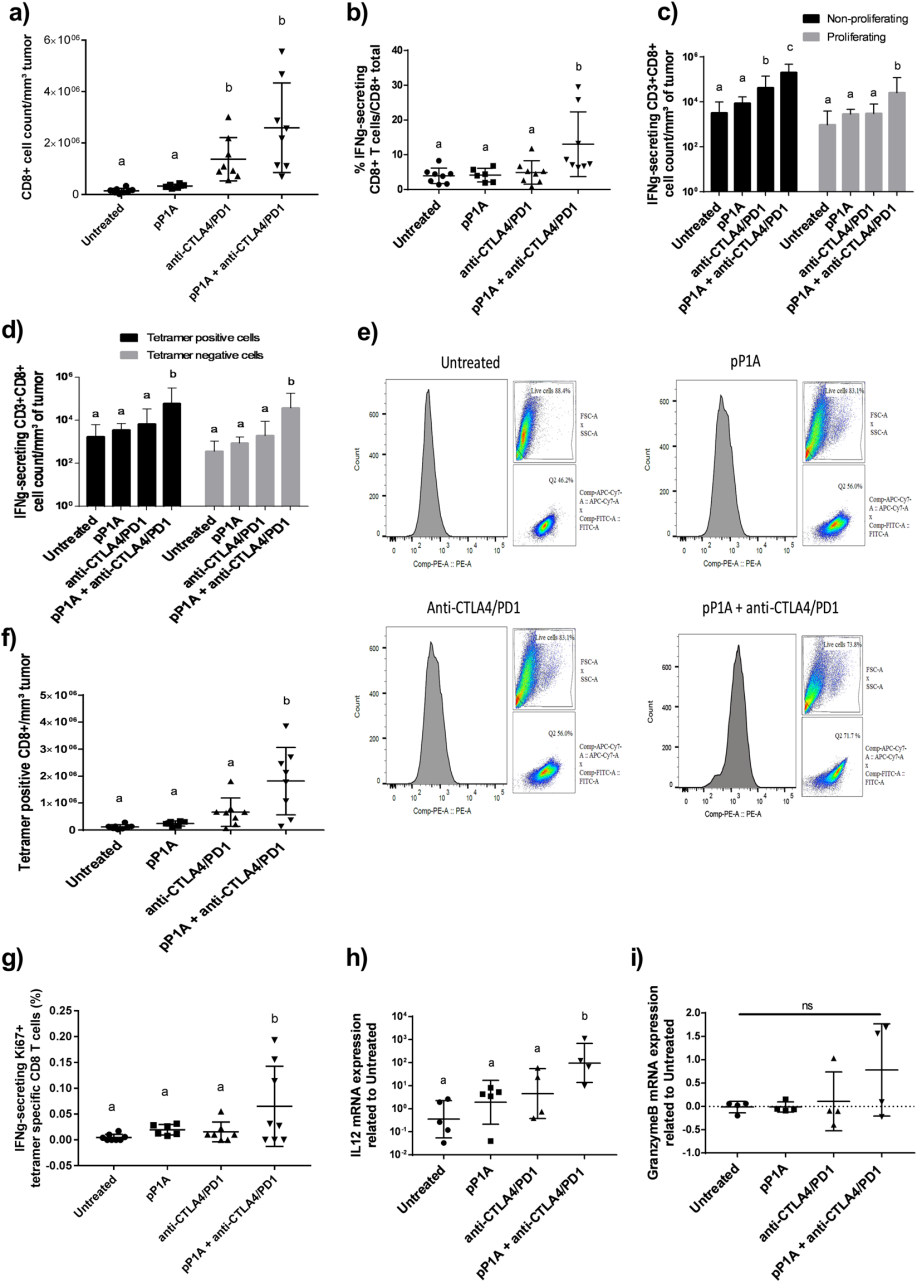
Evaluation of CD8 T cell infiltration, specificity and activity in tumors. (**a**) Number of total CD8 T cells in the tumor (n = 6–8). (**b**) Percentage of IFNg-secreting CD8 T cells compared to the total number of CD3 + CD8 + cells (n = 6–8). (**c**) Number of proliferating (Ki67+) and non-proliferating (Ki67−) IFNg-secreting CD8 T cells per mm³ of tumor (n = 6–8). (**d**) Number of tetramer-positive and -negative IFNg-secreting CD8 T cells per mm³ of tumor (n = 6–8). In (**c**) and (**d**), two independent statistical analyses have been performed for non-proliferating and proliferating cells or for tetramer positive and tetramer negative cells. (**e**) Representative images of flow cytometry analysis for tetramer-specific CD8 T cells in the single tumor cell suspension. Gating strategy: singlets → live cells → CD3 + CD8 + cells → tetramer + cells. (**f**) Antigen-specific CD8 T cells per mm³ of tumor. (**g**) Percentage of P1A antigen-specific IFNg-secreting and proliferating CD8 T cells per mm³ of tumor. (**h**) qPCR analysis of IL12 mRNA expression related to untreated group (n = 4–5). (**i**) qPCR analysis of Granzyme B mRNA expression related to untreated group (n = 4). All results are expressed as the mean ± SD and were considered statistically significant when p < 0.05 (indicated by the lack of common superscript letters) according to one-way ANOVA.

To evaluate the CD8 T cell activity and specific response induced by the vaccine alone or in combination with ICBs, mice were injected with P815 mastocytoma cells. Tumors were removed 10 days later and analyzed by flow cytometry. In general, a greater amount of CD8 T cells was detected in the group treated with ICBs and ICBs + pP1A, which was significantly different compared to the untreated or the pP1A groups (Fig. 3a). Among the CD8 T cells, the number of IFNg-secreting CD8 T cells was significantly higher in the pP1A + anti-CTLA4/PD1 group than in all the other groups (Fig. 3b). In this same group, there was also a significant increase in proliferating IFNg-secreting (Fig. 3c, Supplementary Data 2a) and *P1A* tetramer-specific CD8 T cells (Fig. 3d–g), indicating that the analyzed CD8 T cells were not only antigen specific but also active against tumor cells because of their high secretion levels of IFNg^35^. Furthermore, qPCR analysis of the extracted tumors showed significantly higher IL12 production and a shift in the granzyme B production in the combination group (Fig. 3h,i). IFNg-producing T cells are critical for antitumor immunity^35,36^. The best characterized role of IFNg in CD8 T cell immunity is in enhancing MHC class I antigen presentation pathway, which facilitates cytotoxic T cells to recognize tumor cells. IFNg also enhances CD4 type 1 helper T (Th1) phenotype development^37^, regulates MHC class I expression, has weak cytolytic activity and upregulates the expression of enzymes and cytokines such as Granzyme B and IL12^35^. In particular, the increased production of IL12 observed in the combination-treated mice followed the same trend as IFNg production. Indeed, IL12 initiates or increases IFNg secretion in a positive feedback loop^38^. IL12 also polarizes CD4 T cells into Th1 and regulates the tumor vasculature, thereby playing an important role in tumor rejection^38,39^. IFNg production by antitumor-specific T cells also upregulates PD-L1 on tumor cells as a resistance mechanism to adaptive immunity, thereby promoting PD-L1/PD-1 blockade after vaccination^21^.

The infiltration of antigen-specific CD8 T cells in the group treated with pP1A alone was not significantly different from that in the untreated group. These results suggest that the vaccine alone is not effective in inducing a sufficiently potent immune response in a therapeutic setting due to the immunosuppressive tumor microenvironment, confirming our previous findings^10^. Most likely, the vaccine alone may not be able to ensure sustained CD8 T cell infiltration into the tumor, which could explain the rare presence of antigen-specific CD8 T cells in the tumor. On the other hand, treatment with ICBs alone did not show any significant difference compared to the untreated in priming CD8 T cells against the P815A antigen, but their role in enhancing intratumoral CD8 T cell infiltration has been already demonstrated^3,19^. This complementarity could explain the increased presence of antigen-specific CD8 T cells found in the combination group, as ICBs allow immune cell infiltration, while the vaccine induces their antigen specificity.

### After tumor resection, mice treated with the combination therapy lived longer and without metastases

**Figure 4:**
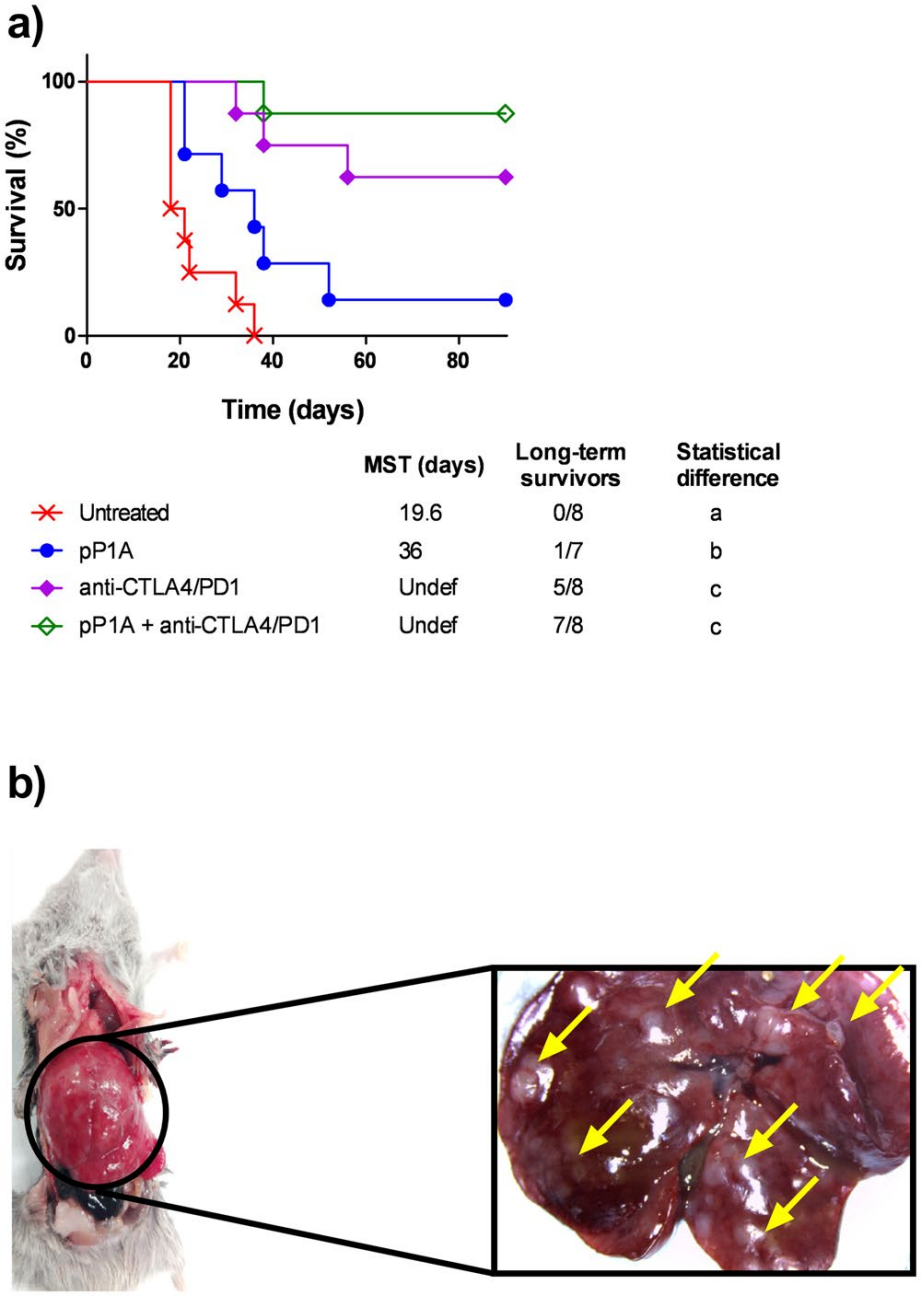
Mouse follow-up after tumor removal at day 10 post-P815 injection. (**a**) Survival curve representing the percentage of living mice (%) as a function of time (days). MST = median survival time. Statistical analysis using log-rank (Mantel-Cox) test to compare each group to the others. Data with no common letters in the statistical analysis (**a**–**c**) are significantly different (p < 0.05, n = 8). (**b**) Representative image of mouse postmortem analysis with magnification of liver metastases.

To evaluate the long-term effect of the treatments, tumors were surgically resected 10 days after tumor injection without sacrificing the mice. As shown in Fig. 4a, 8 days after tumor resection, untreated mice started to die without any visible subcutaneous tumor. Within 3 weeks, all the untreated mice were dead. To understand the reason for their death, postmortem analyses were performed. Their liver was abnormally large and full of metastases (white spots shown in Fig. 4b). The medium weight of these livers was 2.6 ± 0.8 g (n = 4), which was approximately 3 times higher than the weight of a healthy mouse liver. Hence, 10 days after tumor injection, untreated mice had already developed metastasis, and they died shortly later. Mice in the other groups lived longer. As expected, pP1A significantly prolonged mouse survival even when compared to the model without tumor resection (MST = 36 days instead of 29 days, as observed in Figs 1b and 4a), which may be due to a slowdown in metastasis formation induced by the vaccine. This effect has been observed in other studies using cancer vaccines^40,41^ and attests the capacity of these vaccines to act systemically. However, pP1A alone was not sufficient to completely avoid metastasis formation and death. The combination of the vaccine with ICBs significantly prolonged mouse survival compared to the pP1A group, seemingly due to the ICBs blocking metastasis development. A correlation between *PD-L1* and *PD-1* expression and metastasis has been described^42^, which may explain the efficacy of anti-PD1/PDL1 therapy against metastasis. Recently, the role of ICB in restoring the tumor-suppressive capacity of NK cells has also been demonstrated^43^. These cells are involved in the control of metastasis, and their activity depends on the expression of IL12 and Granzyme B, among others^43,44^. Hence, the increased IL12 expression that was observed in this study could explain the metastasis regression and the higher survival in the ICB and ICB + pP1A groups.

## Conclusions

This study evaluates the main limitations of the use of DNA vaccines and ICB as single treatment against cancer. It also analyses the reasons of the successful combination of these two therapies, giving a future perspective on how to optimize anticancer immunotherapies. CTLA4 and PD1 antagonists have been widely used in preclinical and clinical trials due to their ability to enhance tumor-reactive T-cell responses^45,46^. However, they are not able to specifically prime T cells and they are toxic in the majority of the patients^47^.

In this study, the combination of ICBs and DNA vaccination permitted to decrease the doses of the antibodies and generated a more potent immune response compared to the single therapies. Their effects in the tumor microenvironment were analyzed in a very early phase of tumor development, i.e., 10 days after the tumor injection, which was shown to be a critical stage for metastases formation. This synergy was due to the ability of DNA vaccination to prime T cells against a specific cancer antigen and of ICBs to increase T cell activity and infiltration in the tumor. Hence, ICBs created a favorable microenvironment for the action of the cancer DNA vaccine. Both the pP1A vaccine and ICB as single therapies showed an increase in CD4 T cells. ICB significantly increased also the number of IFNg-secreting CD8. When used in combination, however, pP1A and ICBs significantly increased IFNg and IL12 production but also CD4 and CD8 T cell infiltration 10 days after tumor implantation. This result explains the significant delay in tumor growth among mice treated with pP1A alone, ICB alone or pP1A + ICB, demonstrating an improved outcome in response to the combination therapy.

Compared to the untreated group, the group treated with ICBs and pP1A showed a protective effect when the tumors were removed, as they retarded the formation of liver metastases appearing in the early phase of tumor growth. These results indicated that immune activation starts to appear in the early phase of tumor development, especially when the combination therapy of the DNA vaccine and ICBs is used.

Overall, this study suggests and supports the idea of a rational combination of cancer DNA vaccines able to generate a tumor-specific and long-lasting immune response with therapies that increase immune cell infiltration, proliferation and activity in the tumor, such as ICB. In addition, it demonstrated that the beneficial effects of DNA vaccine in combination with ICBs appeared as soon as one week after administration, allowing 7 out of 8 mice to survive after a tumor injection and avoiding metastases formation.

## Materials and Methods

### Plasmid optimization and production

The P1A DNA vaccine encoding the P1A antigen gene (here named plasmid P1A, pP1A) has been previously described^10^. The plasmid was amplified and purified using an EndoFree Plasmid Giga Kit (Qiagen, Venlo, NL) according to the manufacturer’s protocol. Optical density at 260 nm was used to determine DNA concentration. Plasmid was diluted in phosphate buffered saline (PBS, Thermo Fisher, Waltham, Massachusetts, USA) and stored at −20 °C before use.

### Cell lines

*P1A*-expressing P815 murine cells were obtained from Dr. Catherine Uyttenhove (Ludwig Institute for Cancer Research) and cultured at 37 °C in 5% CO_2_ in Dulbecco’s modified Eagle’s medium (DMEM, Thermo Fisher, Waltham, Massachusetts, USA) supplemented with 10% fetal bovine serum (FBS, Thermo Fisher, Waltham, Massachusetts, USA) and 1% penicillin/streptomycin (Thermo Fisher, Waltham, Massachusetts, USA).

### Animals

DBA/2 female mice were obtained from Janvier (Le Genest-Saint-Isle, FR). Mice were between 5 and 6 weeks old at the beginning of the experiments. Water and food were provided ad libitum. All experimental protocols using mice were approved by the Ethical Committee for Animal Care and Use of the Medical Sector of the Université Catholique de Louvain (UCL/MD/2011/007 and UCL/MD/2016/001). All experiments were performed in accordance with relevant guidelines and regulations.

### Tumor implantation and tumor growth measurement

At day 0, 1 × 10^6^ P815 cells diluted in 100 μl of PBS were injected subcutaneously into the right flank of each mouse. Tumors were measured with an electronic digital caliper three times per week. Tumor volumes were calculated as length × width × height (in mm^3^). Mice were sacrificed when the tumor volume was greater than 1500 mm^3^ or when they were in poor condition and expected to die shortly. Tumors were collected and used for further experiments.

### Therapeutic vaccinations and ICB administration

Before each vaccine injection, mice were anesthetized with ± 150 μl of a solution of 10 mg/ml ketamine (Ketalar, Pfizer, New York, USA) and 1 mg/ml xylazine (Sigma, St. Louis, MO, USA). The left paw was shaved using a rodent shaver (Aesculap Exacta shaver, AgnTho’s, Stockholm, SE). Mice in the pP1A (n = 10) and pP1A + anti-CTLA4/PD1 (n = 10) groups were injected with 50 µg of pP1A diluted in 30 µl of PBS in the left tibialis cranial muscle. The paw was then placed between 4 mm plate BTX caliper electrodes (VWR International, Leuven, BE) and electroporated (200 V/cm, 8 pulses, 20 ms with 500 ms pause between pulses). The pulses were delivered by a BTX™ Gemini Electroporation System (VWR International, Leuven, BE). The vaccine was administered 2, 9 and 16 days after tumor injection. Immune checkpoint blockade (ICB) antibodies directed against CTLA4 (clone 9D9) and PD1 (clone 29 F.A12) were purchased from Bioconnect (Huissen, NL). Mice in the anti-CTLA4/PD1 and pP1A + anti-CTLA4/PD1 (n = 10) groups were injected intraperitoneally (IP) with 100 µg of each antibody in 100 µl of PBS 3, 6 and 9 days after tumor injection. Nonimmunized mice were used as a negative control (n = 10). The protocol is presented in Fig. 1.

### CD4 and CD8 T cell detection by flow cytometry

For the detection of CD4 and CD8 T cells in the tumor microenvironment, tumors were surgically removed, and tumor volume was measured 10 days after P815 cell injection. To prepare single cell suspensions, tumors were digested for 1 hour in 1 mg/ml collagenase type II (Sigma, Saint-Louis, Missouri, USA). Cells were collected, counted using an automatic cell counter (BioRad, California, USA) and washed with PBS containing 5 mM EDTA and 1% albumin. Cells were then incubated with an anti-CD16/CD32 antibody for 10 minutes on ice (clone cl93, Biolegend, San Diego, California, USA). Cells were washed and incubated for 60 minutes at 4 °C with a cocktail of the following antibodies: anti-CD3-APC-Cy7 (clone 17A2, Biolegend, San Diego, California, USA), anti-CD4-FITC (clone GK1,5, Biolegend, San Diego, California, USA), anti-CD8-FITC (clone 53-6,7, Biolegend, San Diego, California, USA) and P1A tetramers-phycoerythrin (PE). For staining with anti-Ki67-BV421 (clone B56, BD Bioscience, Franklin Lakes, New Jersey, USA), antiFoxP3-APC (clone FJK-16s, Thermo Fisher, Waltham, Massachusetts, USA) and anti-IFNg-APC (clone XMG1.2, Biolegend, San Diego, California, USA), cells were previously incubated overnight at 4 °C with a permeabilization/fixation solution (eBioscience™ Foxp3/Transcription Factor Staining Buffer Set, Thermo Fisher, Waltham, Massachusetts, USA). Cells were then incubated with anti-CD16/CD32 antibody for 10 minutes on ice (Biolegend, San Diego, California, USA). Cells were washed and incubated for 60 minutes at 4 °C with two different cocktails of antibodies diluted in the permeabilization/fixation solution: anti-Ki67, anti-IFNg (first cocktail) and anti-Ki67 and anti-FoxP3 (second cocktail). Samples were washed with PBS containing 5 mM EDTA and 1% albumin, and the cells were then suspended in PBS. For each staining panel, a negative control (non-stained cells) was performed. For each antibody, we performed a control with cells stained with one antibody at the time. As the mice were sacrificed 24 h after the last vaccine and ICB injection and tumors analyzed right after the sacrifice, no stimulation was needed to test the production of IFNg^48^. Sample data were acquired with FACSverse (BD bioscience, Franklin Lakes, New Jersey, USA) and analyzed with FlowJo software (FlowJo LLC, Ashland, OR, USA). The number of cells was normalized by the tumor volume (mm³).

### Determination of Granzyme B and IL12 expression in tumors using qPCR analysis

To further determine the activity of immune cells present in the tumor microenvironment, tumors extracted at day 10 were analyzed by qPCR. Total RNA was isolated using TRIzol reagent (Thermo Fisher, Waltham, Massachusetts, USA) and phenol separation. The quality and quantity of RNA were evaluated using a nanospectrophotometer (NanoDrop 2000, Thermo Fisher Scientific). Extracted RNA was considered pure if the 260/280 absorbance ratio of the sample was approximately 2 and the 260/230 absorbance ratio was 1.8–2.2. One microgram of RNA was reverse transcribed using a first-strand synthesis system (SuperScript^TM^, Thermo Fisher Scientific) and oligo(dT) primers (Eurogentec, Liege, BE) according to the supplier’s protocol. The resulting cDNA was used as template for 40 cycles of PCR amplification. SYBR™ green real-time qPCR (GoTaq qPCR MasterMix kit, Promega, Fitchburg, WI, USA) was conducted on a StepOne Plus Real-Time PCR System (Thermo Fisher Scientific) to detect IL12 and Granzyme B mRNA expression in the tumors. Analysis of the melting curves was performed to ensure purity of PCR products. The results were analyzed with StepOne Software V2.1. The mRNA expression of the cytokines was calculated relative to the corresponding expression of β-actin (housekeeping gene) according to the delta-delta Ct method. The results were normalized compared to the untreated control group. Primers for IL12 and Granzyme B were designed using Primer Blast software based on the consensus of sequences from GenBank. To determine PD-L1 mRNA expression, RNA from tumors at day 10 and from P815 cells was extracted and converted into cDNA as described previously. The resulting cDNA was used as a template for 30 cycles of semiquantitative PCR with a T100 thermocycler (Bio-Rad). Primers for β-actin (housekeeping gene) and PD-L1 were used to amplify respective cDNA by PCR. The PCR products were subjected to electrophoresis on a SYBR Safe (Thermo Fisher Scientific)-stained 1% agarose gel. A 1 bk DNA ladder (Sigma, Saint Louis, Missouri, USA) was used to check the length of the amplicon. A complete list of the primers used in this study is shown in Table 1.

**Table 1:** List of primers used for qPCR analysis.

Oligo name	Primer sequence (5′ → 3′)	Amplicon length
Granzyme B for	GAAGCCAGGAGATGTGTGCT	183 bp
Granzyme B rev	GCACGTTTGGTCTTTGGGTC	
IL-12 for	GGAAGCACGGCAGCAGAATA	180 bp
IL-12 rev	AACTTGAGGGAGAAGTAGGAATGG	
β-actin for	ACTCCTATGTGGGTGACGAG	206 bp
β-actin rev	CATCTTTTCACGGTTGGCCTTAG	
PD-L1 for	TAATCAGCTACGGTGGTGCG	273 bp
PD-L1 rev	AAACATCATTCGCTGTGGCG	

### Statistical analysis

Statistical analyses were performed using GraphPad Prism 7 for Windows. Survival curves were compared using a Mantel–Cox (log-rank) test. p-Values less than 0.05 were considered statistically significant. Data with no common superscript letter (a, b, c) were considered significantly different (p < 0.05) according to ANOVA.

## Electronic supplementary material

Dataset 1

Dataset 2
