# Supplementary material for: Combination of immune checkpoint blockade with DNA cancer vaccine induces potent antitumor immunity against P815 mastocytoma
Source: Sci Rep. 2018 Oct 24;8:15732. doi: 10.1038/s41598-018-33933-7 (PMC6200811; doi:10.1038/s41598-018-33933-7)
Supplement: Supplementary file 1 — Dataset 1 [file 41598_2018_33933_MOESM1_ESM.pdf]

Combination of immune checkpoint blockade with DNA cancer vaccine induces potent antitumor immunity against P815 mastocytoma

Alessandra Lopes[1], Kevin Vanvarenberg[1], Špela Kos[2], Sophie Lucas[3], Didier Colau[3,4], Benoît Van den Eynde[3,4], Véronique Préat[1*#], Gaëlle Vandermeulen[1#]

Supplementary data 1

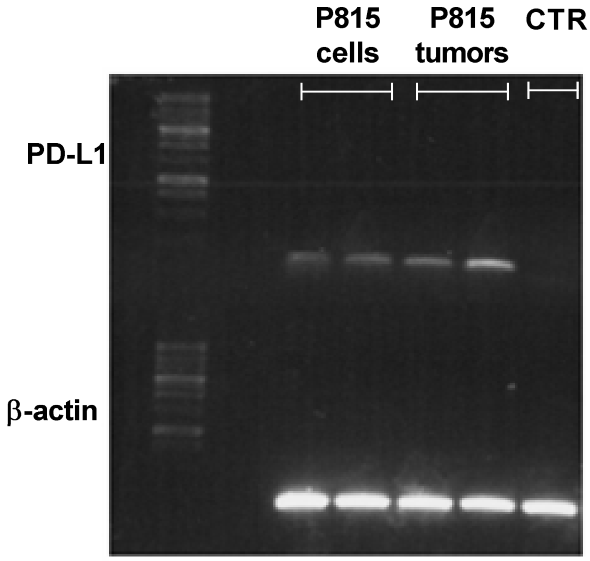

RT-PCR of PD-L1 mRNA expression in P815 cells and untreated tumors (n = 2). Control group is a sample without the PD-L1 primers (CTR).
