# Supplementary material for: Combination of immune checkpoint blockade with DNA cancer vaccine induces potent antitumor immunity against P815 mastocytoma
Source: Sci Rep. 2018 Oct 24;8:15732. doi: 10.1038/s41598-018-33933-7 (PMC6200811; doi:10.1038/s41598-018-33933-7)
Supplement: Supplementary file 2 — Dataset 2 [file 41598_2018_33933_MOESM2_ESM.pdf]

Combination of immune checkpoint blockade with DNA cancer vaccine induces potent antitumor immunity against P815 mastocytoma

Alessandra Lopes[1], Kevin Vanvarenberg[1], Špela Kos[2], Sophie Lucas[3], Didier Colau[3,4], Benoît Van den Eynde[3,4], Véronique Préat[1*#], Gaëlle Vandermeulen[1#]

Supplementary data 2

a) IFNg-secreting and proliferating CD8 T cells

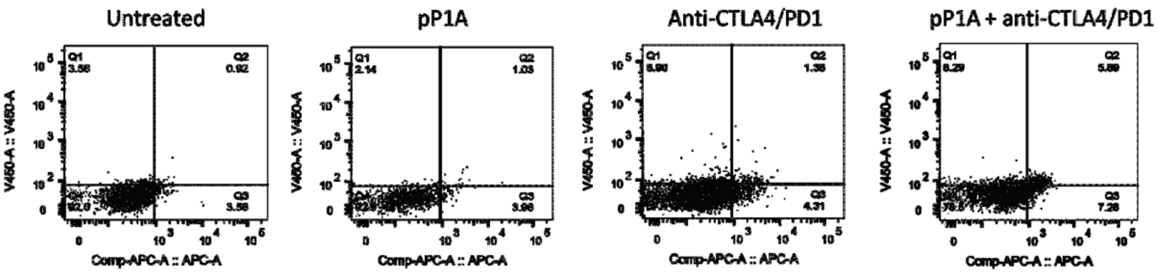

b) CD4+FoxP3- proliferating T cells

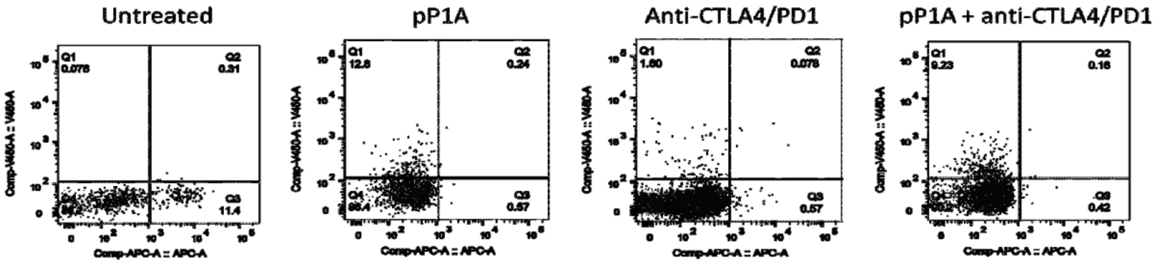

a) IFNg-secreating and proliferating CD8.

The gating strategy used was as follow: singlets → live cells → CD3+CD8+ cells → Ki67-IFNg cells

b) CD4+FoxP3- proliferating T cells.

The gating strategy used was as follow: singlets → live cells →CD3+CD4+ cells → FoxP3-Ki67
